# Do blood flow patterns in the left atriums differ between left upper lobectomy and other lobectomies? A computational study

**DOI:** 10.3389/fcvm.2023.1305526

**Published:** 2024-01-05

**Authors:** Wentao Yi, Tomohiro Otani, Shunsuke Endo, Shigeo Wada

**Affiliations:** ^1^ Department of Mechanical Science and Bioengineering, Graduate School of Engineering Science, Osaka University, Osaka, Japan; ^2^ Saitama Medical Center, Jichi Medical University, Saitama, Japan

**Keywords:** left atrium, lobectomy, pulmonary vein, computational fluid dynamics, hemodynamics

## Abstract

**Background:**

Left atrial (LA) hemodynamics after lung lobectomies with pulmonary vein (PV) resection is widely understood to be a risk factor for LA thrombosis. A recent magnetic resonance imaging study showed that left upper lobectomy (LUL) with left superior pulmonary vein resection tended to cause LA flow patterns distinct from those of other lobectomies, with flow disturbances seen near the PV stump. However, little is known about this flow pattern because of severe image resolution limitations. The present study compared flow patterns in the LA after LUL with the flow patterns of other lobectomies using computational simulations.

**Methods:**

The computational simulations of LA blood flow were conducted on the basis of four-dimensional computed tomography images of four lung cancer patients prior to lobectomies. Four kinds of PV resection cases were constructed by cutting each one of the PVs from the LA of each patient. We performed a total of five cases (pre-resection case and four PV resection cases) in each patient and evaluated global flow patterns formed by the remaining PV inflow, especially in the upper LA region.

**Results:**

LUL tended to enhance the remaining left inferior PV inflow, with impingements seen in the right PV inflows in the upper LA region near the PV stump. These flow alterations induced viscous dissipation and the LUL cases had the highest values compared to other PV resection cases, especially in the LV systole in three patients, and reached three to four times higher than those in pre-resection cases. However, in another patient, these tendencies were weaker when PV inflow was stronger from the right side than from the left side, and the degree of flow dissipation was lower than those in other PV resection cases.

**Conclusion:**

These findings suggest marked variations in LA flow patterns among patients after lobectomies and highlights the importance of patient-specific assessment of LA hemodynamics after lobectomies.

## 1 Introduction

Left atrial (LA) thrombosis has received much attention as a cause of cerebral infarction. Although LA thrombosis is thought to occur in pathological conditions such as LA fibrillation (1), recent cohort studies have shown that lung lobectomy, which is a common surgical treatment for lung cancer patients (2), is a significant risk factor for LA thrombosis even in patients with no prior history of cardiac disease (3–5). A lobectomy requires an associated pulmonary vein (PV) resection from the LA and thrombus formation in the PV stump, where the remaining PV resected, was reported for 3.6% of 193 patients (6). Several potential factors of thrombus formation have been proposed such as LA hemodynamic alteration (7–11), PV stump morphologies (7), and inflammatory responses (12). However, thrombus formation after lobectomy is rare (approximately 5% of LUL cases (5)), and the mechanism of the thrombosis in the PV stump therefore remains unclear.

Furthermore, several clinical studies reported that left upper lobectomy (LUL) with left superior PV (LSPV) resection has a relatively high risk of thrombus formation (5, 6, 13), while this causal mechanism is unknown. To clarify the characteristic differences of the LUL from viewpoints of LA hemodynamics, Umehara et al. (14) compared LA flow patterns among four types of lobectomies, i.e., LUL and left-lower, right-upper, and right-lower lobectomies regardless of the thrombus formation by four-dimensional flow magnetic resonance imaging (MRI). They reported that LUL likely caused flow disturbances around the LSPV stump, which was absent in almost all cases treated with the other types of lobectomies. However, these findings were based on flow pathlines derived from flow velocity fields on MRI scans with severely limited spatiotemporal resolution. These LA flow patterns were variable and complex, characterized by multiple intermixing PV inflows, and little is known about them given the limited MRI data.

To address this issue, computational fluid dynamics (CFD) simulation of LA hemodynamics is a promising approach to describe flow patterns after lobectomies and associated PV resections. We previously investigated the possible effects of LUL (15) and associated physiological changes (16) on LA hemodynamics, and found remarkable LA flow changes associated with left and right PV inflow impingement after LUL. Because the computational approach can represent lobectomies with PV resection virtually in each patient, CFD simulations of LA hemodynamics with virtual PV resection could reveal the distinctive LA flow patterns after LUL implied by MRI studies through comparison with those after other lobectomies.

This study investigated LA flow patterns after different types of lobectomies using four-dimensional computed tomography (4D-CT)-based computational simulations and analyzed distinctive flow patterns after LUL. Following CFD simulation of LA blood flow and virtual PV resections, as developed in our previous study (16), we compared LA blood flow patterns before and after PV resection in four patients.

## 2 Material and methods

This study conducted CFD simulations of LA hemodynamics using 4D-CT images of four lung cancer patients before lobectomy. They received an LUL at the Department of Thoracic and Cardiovascular Surgery at Jichi Medical University Hospital. Thrombus formation in the PV stump was not found for each patient after the LUL. CT images were acquired in previous studies (15, 16). The study was approved by the Institutional Review Board of Jichi Medical University.

### 2.1 Preprocessing

First, 4D-CT images of four lung cancer patients (participants 1–4) were obtained using a 128-slice multi-detector CT scanner (SOMATOM Definition Flash; Siemens, Berlin, Germany) at Jichi Medical University Hospital and reconstructed over 20 phases of the cardiac cycle from ventricular end-diastole. The cardiac length T in each participant was shown in Table 1.

**Table 1:** Participant information: cardiac length T (ms) and cross-sectional area (mm${}^{2}$) of each pulmonary vein (PV) in each participant (left superior PV: LSPV, left inferior PV: LIPV, right superior PV: RSPV, right inferior PV: RIPV).

	Cardiac cycle (ms)	Cross-sectional area (mm${}^{2}$)			
		LSPV	LIPV	RSPV	RIPV
Subject 1	870	211	97.2	194	212
Subject 2	920	271	225	271	145
Subject 3	1000	180	116	196	145
Subject 4	984	95.3	171	175	135

**Figure 1:**
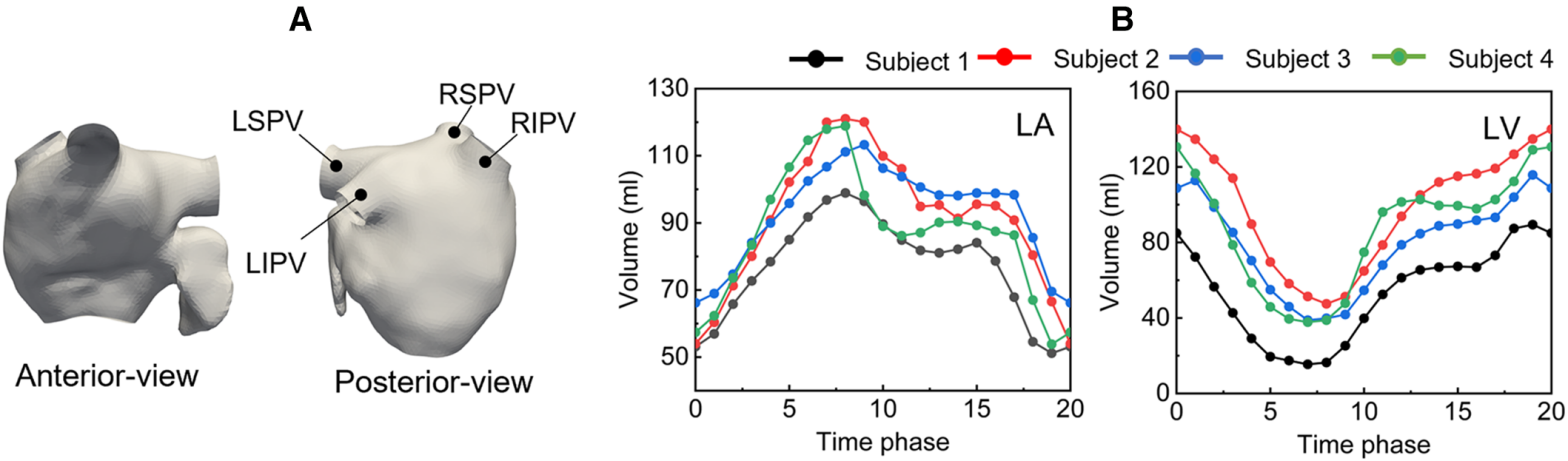
Representative left atrium surface (anterior and posterior views) of participant 1 at phase 9(a), and time courses of the left atrium and left ventricle volume (b).

LA surface shapes in each phase were reconstructed using Mimics software (version 23; Materialise, Inc., Yokohama, Japan), in which cuts were made in proximal parts of the PVs before the first bifurcation, where the PV cross section was orthogonal to the PV direction. Figure 1 shows a representative LA surface (a) and time courses of the LA and left ventricle (LV) volume in each participant (b). The PV cross-sectional area is shown in Table 1. In each patient, the cross-sectional areas of the left and right superior PVs (LSPV and RSPV, respectively) tended to be larger than those of the left and right inferior PVs (LIPV and RIPV, respectively), except for the LSPV in participant 4.

LA wall displacements in a cardiac cycle were estimated from non-rigid point registration (17, 18) and temporally interpolated by Fourier series expansion. Cylindrical tubes were attached to sections of the PVs and mitral valve (MV) to reduce the effects of artificially set boundary conditions on LA blood flow (19). Furthermore, the time courses of LV volume were interpolated using Fourier series expansion to compute the total flow rate through the MV in LV diastole.

#### 2.1.1 Virtual PV resection

**Figure 2:**
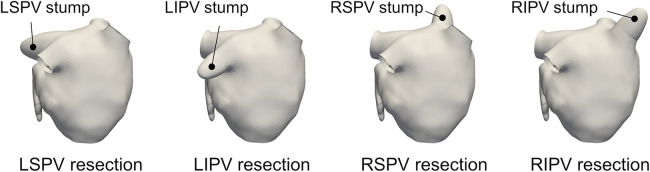
Representative left atrium surfaces with virtual pulmonary vein (PV) resections of the left-superior (LS), left-inferior (LI), right-superior (RS), and right-inferior (RI) PVs for participant 1.

To compare LA blood flow patterns with different PV resections, each PV resection case was constructed for each subject, as shown in Figure 2. Each one of the PVs was cut and smoothly filling the incision to form a stump-like structure as in Yi et al. (16). Consequently, a total of five cases (one pre-cases and four PV-resection cases) was prepared in each participant.

### 2.2 Computational fluid dynamics simulation

In the CFD simulations, LA blood flow was treated as a prescribed moving-wall boundary problem using moving LA surfaces. The simulations were performed using a Cartesian grid CFD solver developed in (15, 20). Blood was assumed to be an incompressible Newtonian fluid, and continuity and incompressible Navier-Stokes equations were adopted to yield the governing equation:

(1) ∇⋅v=0,

(2) $$\rho (\partial_{t}v+v\cdot \nabla v)=-\nabla p+\eta \nabla^{2}v,$$

where v is the velocity vector, p is pressure, blood density $\rho =1.05\times 10^{3}$ kg/m${}^{3}$, and dynamic viscosity $\eta =3.5\times 10^{-3}$ Pa⋅s. Governing equations are discretized using the finite difference method in conventional staggered grids, and flow velocities and pressure are updated in a step-by-step manner. For details of the computational schemes and spatiotemporal discretization, please see our previous work (15).

The boundary conditions are as follows. The no-slip boundary condition was assigned on the wall boundary using the boundary data immersion method (21). The MV was assumed to be a wall boundary when it was closed during LV systole, whereas a uniform transient velocity profile was set at the MV when the MV opened during LV diastole. The MV flow rate profile was determined by the time course of the LV volume change shown in Figure 1B. On each PV cross-section, we applied uniform pressure boundary conditions of pressure $P_{PV}$, expressed as $P_{PV}=P_{0}+K_{i}Q_{PV}$, where $P_{0}$ is the baseline, $K_{i}$ is the terminal resistance at each PV, and $Q_{PV}$ is the flux through the PV cross section. We set $P_{0}=0$ Pa and $K_{i}$ to be inversely proportional to the corresponding cross-sectional area of each PV, assuming that the velocity was the same for all PVs (22).

The computational domain was discretized as uniform Cartesian grids of 256×256×256, where the grid size was 0.6 mm in each direction in accordance with a test of grid size dependencies conducted in our previous study (15). The time increment was chosen to ensure that the Courant–Friedrichs–Lewy number was less than 0.1. Each computational simulation was conducted over three cardiac cycles, and the Results section shows the solution in the third cycle unless otherwise noted.

### 2.3 Postprocessing

To clarify the global LA blood flow pattern in each PV resection case from a fluid mechanical sense, we adopted Lagrange-based flow descriptors which extract flow characteristics from flow velocity fields. First, the flow pathline from 0T to 0.35T was depicted by mass-less tracers $x_{p}$ set in each PV section before PV resection to confirm the PV inflow directions. Next, backward finite-time Lyapunov exponents (FTLEs) in the cross-sectional plane of the upper LA region were visualized in all cases to capture PV inflow before and after each PV resection. The backward FTLE can be understood as Lagrange coherent structure, which is locally the most strongly attracting material surface (i.e., flow boundaries) according to dynamical systems theory (23, 24), and thus is effective in quantifying complex LA flow topologies created by multiple PV inflows. The FTLEs were computed from the flow map $\phi_{t_{0}}^{t_{0}+\tau }\;:\;x_{p}(t_{0})\Rightarrow x_{p}(t_{0}+\tau )$ with respect to mass-less tracers at initial time $t_{0}$ by solving the advection in the LA blood flow in finite time τ (23), given by

(3) $$\text{FTLE}=\frac{1}{\left|\tau \right|}\ln{\left\|\frac{\partial \phi_{t_{0}}^{t_{0}+\tau }}{\partial x}\right\|}_{2}.$$

Here, We adopted τ=50 ms. For negative τ (<0), the FTLE ridges show directions of high flow stretching, known as attracting Lagrange coherent structure. Trajectories of mass-less tracers were computed by fourth-order Runge-Kutta method, and flow velocity fields were linearly interpolated in space and time in each grid.

Furthermore, global flow characteristics in the LA were evaluated on the basis of the flow kinetic energy and dissipation rate, given by

(4) $$\text{Kinetic\textbackslash{} energy}=\int_{\Omega_{f}}\frac{1}{2}\rho (v\cdot v)d\Omega ,$$

(5) $$\text{Dissipation\textbackslash{} rate}=\int_{\Omega_{f}}2\eta S\;:\;Sd\Omega ,$$

where $\Omega_{f}$ is the LA domain and S indicates the symmetric components of ∇v.

## 3 Results

### 3.1 Flow pathlines

**Figure 3:**
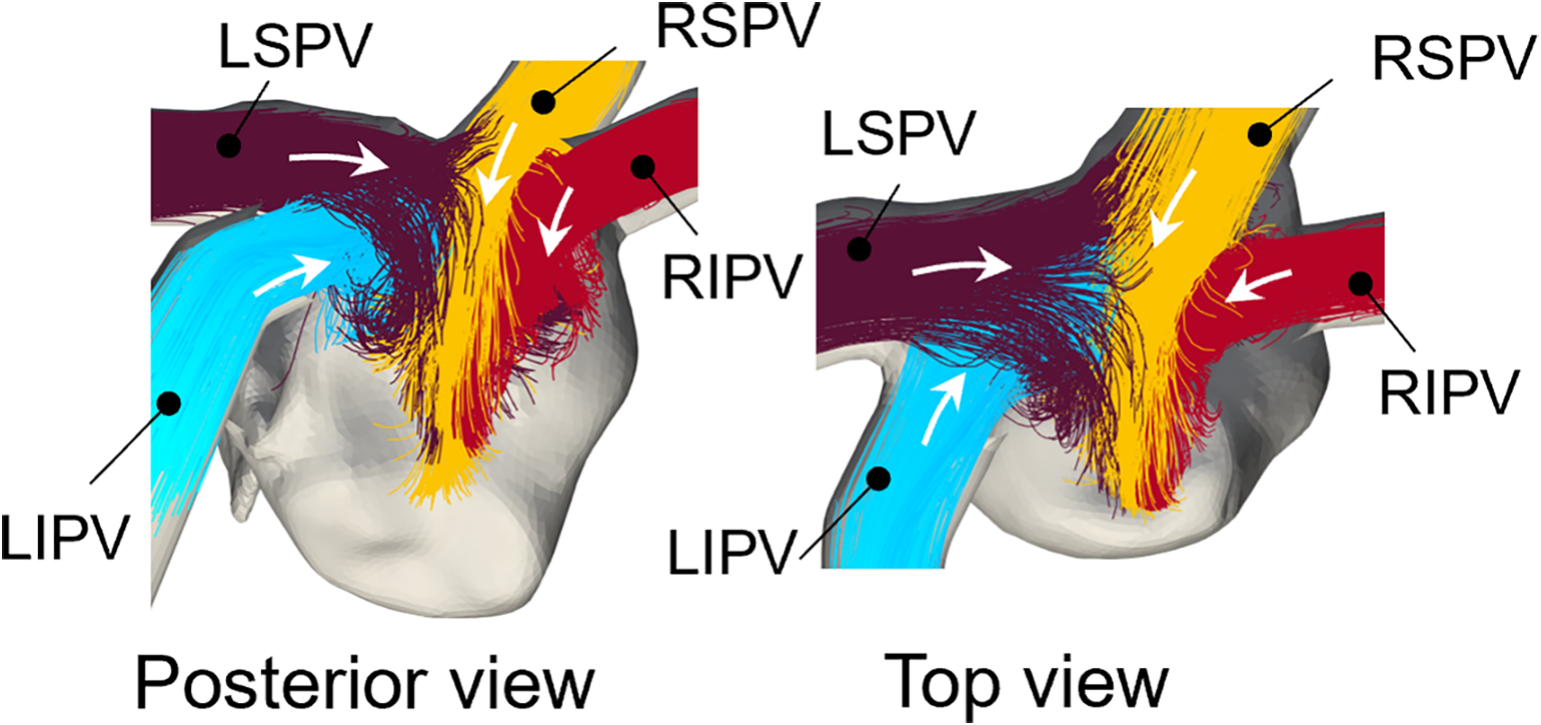
Representative pre-resection flow pathlines from 0T to 0.35T for participant 2 (LSPV: left superior pulmonary vein, LIPV: Left inferior pulmonary vein, RSPV: right superior pulmonary vein, RIPV: right inferior pulmonary vein). Colors of pathlines show each PV inflow and white arrows show the inflow direction from the pulmonary veins.

Figure 3 shows representative flow pathlines of participant 2 before the lobectomies. LSPV inflow entered along the LA roof and LIPV inflows entered underneath the LSPV inflow. The inflows from the right PVs flowed directly into the inferior region of the LA, almost in parallel. These characteristics were also observed in other participants.

**Figure 4:**
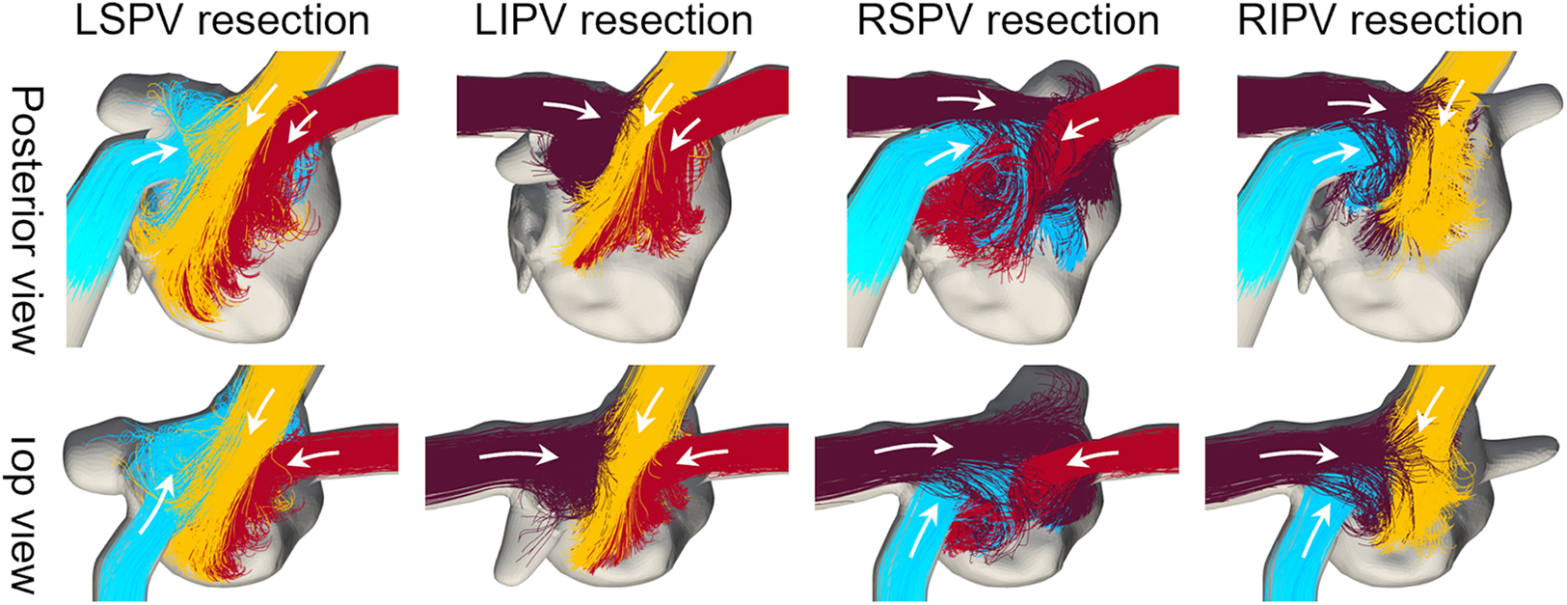
Same as Figure 3, but in cases with virtual PV resections.

The flow pathlines in each virtual PV resection are shown in Figure 4. In LSPV resections, LIPV inflows entered directly, flowed toward the anterior LA wall, and impinged on the RSPV inflows. In RSPV resections, LSPV inflows also reached the right side of the LA and flowed downward. The changes in the remaining inflow patterns were relatively small in LIPV and RIPV resections.

### 3.2 Backward finite-time Lyapunov exponents

**Figure 5:**
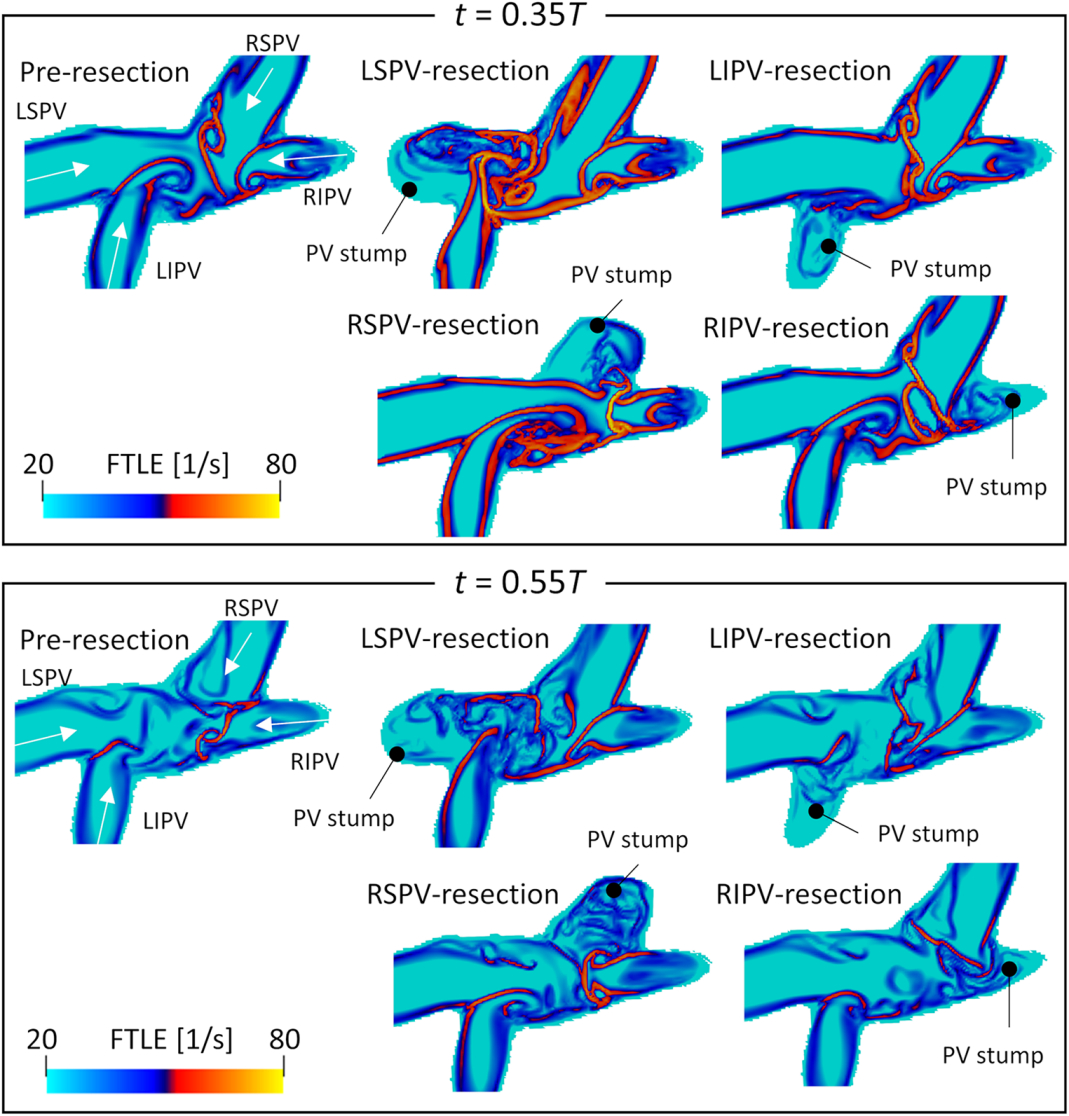
Spatial distribution of the backward finite-time Lyapunov exponent on the cross-sectional plane of the left atrial upper region, at t=0.35T (systole) and t=0.55T (diastole) in participant 2 before PV resection (LSPV, left superior pulmonary vein; LIPV, left inferior pulmonary vein; RSPV, right superior pulmonary vein; RIPV, right inferior pulmonary vein). Representative PV resection cases are also shown. These time courses are shown in a whole cardiac cycle in Supplementary Video 1.

Figure 5 shows the backward FTLEs in the cross-sectional plane of the upper LA regions before PV resection, as well as each PV resection at end-systole (t=0.35T) and early-diastole (t=0.55T). Supplementary Video 1 shows time courses of FTLEs in all virtual PV resection cases over one cardiac cycle. Here, boundaries between PV inflows are shown in a high-FTLE region (attracting Lagrange coherent structure). In the LSPV resection, flow impingement was found between LIPV and RSPV inflows, and flow disturbances occurred near the LSPV stump. In the RSPV resection, the LSPV inflows reached the right side of the LA, while flow impingement was not found in the upper LA region and flow in the RSPV stump was isolated. In LIPV and RIPV resections, FTLE distributions generated by remaining PV inflows were almost the same as those in pre-resection cases.

**Figure 6:**
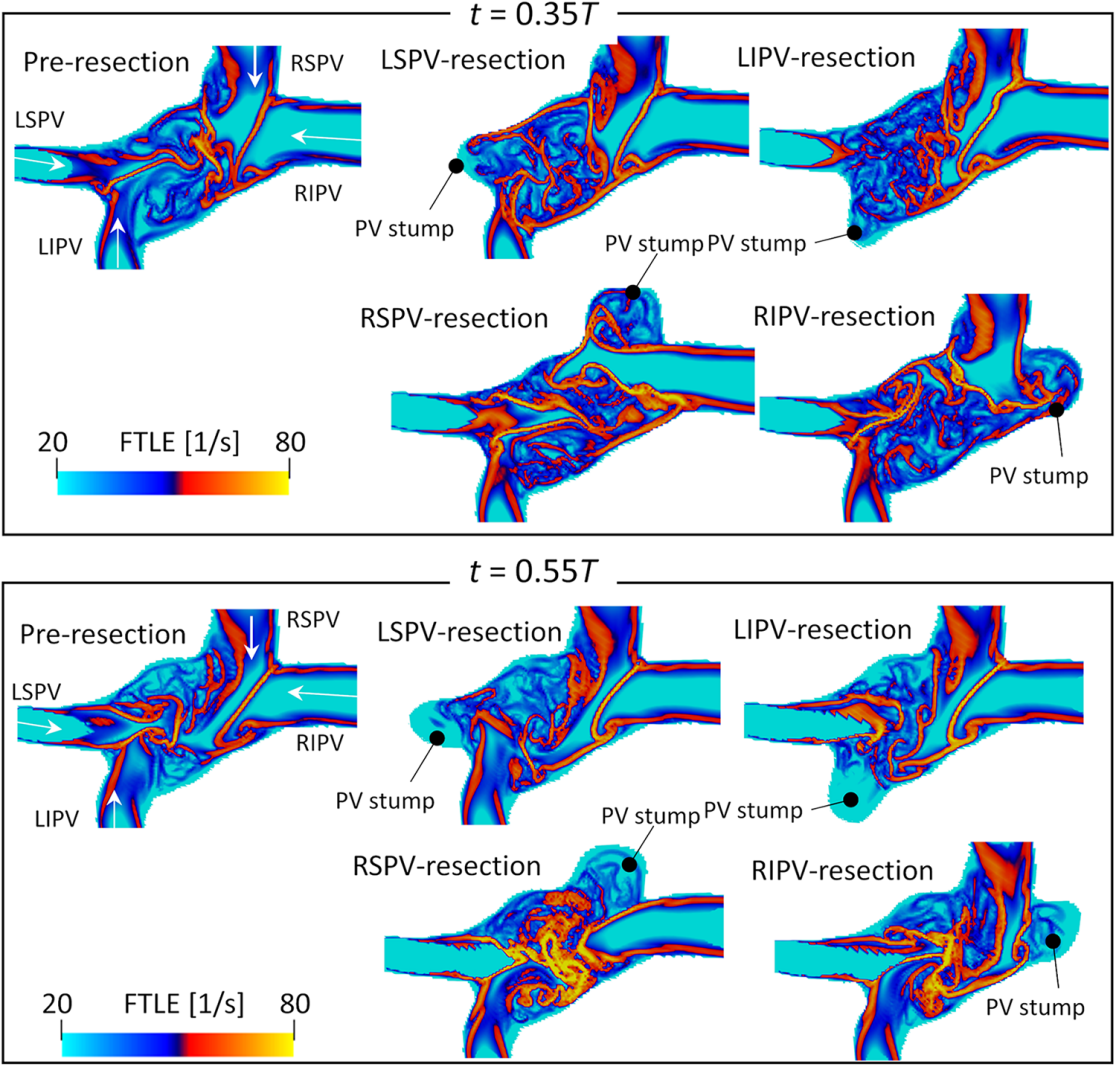
Same as Figure 5 but for participant 4.

Figure 6 shows the backward FTLE distribution in participant 4 at end-systole (t=0.35T) and early-diastole (t=0.55T). Both LSPV and LIPV resection caused flow disturbances in the center region, whereas the flow boundaries of the right PV inflows were almost the same as the pre-resection ones. Conversely, RSPV resection enhanced the RIPV inflows, which extended to the anterior side of the LA. Similarly, in the RIPV resection case, RSPV inflows entered the posterior side of the LA.

### 3.3 Global energetic states

**Figure 7:**
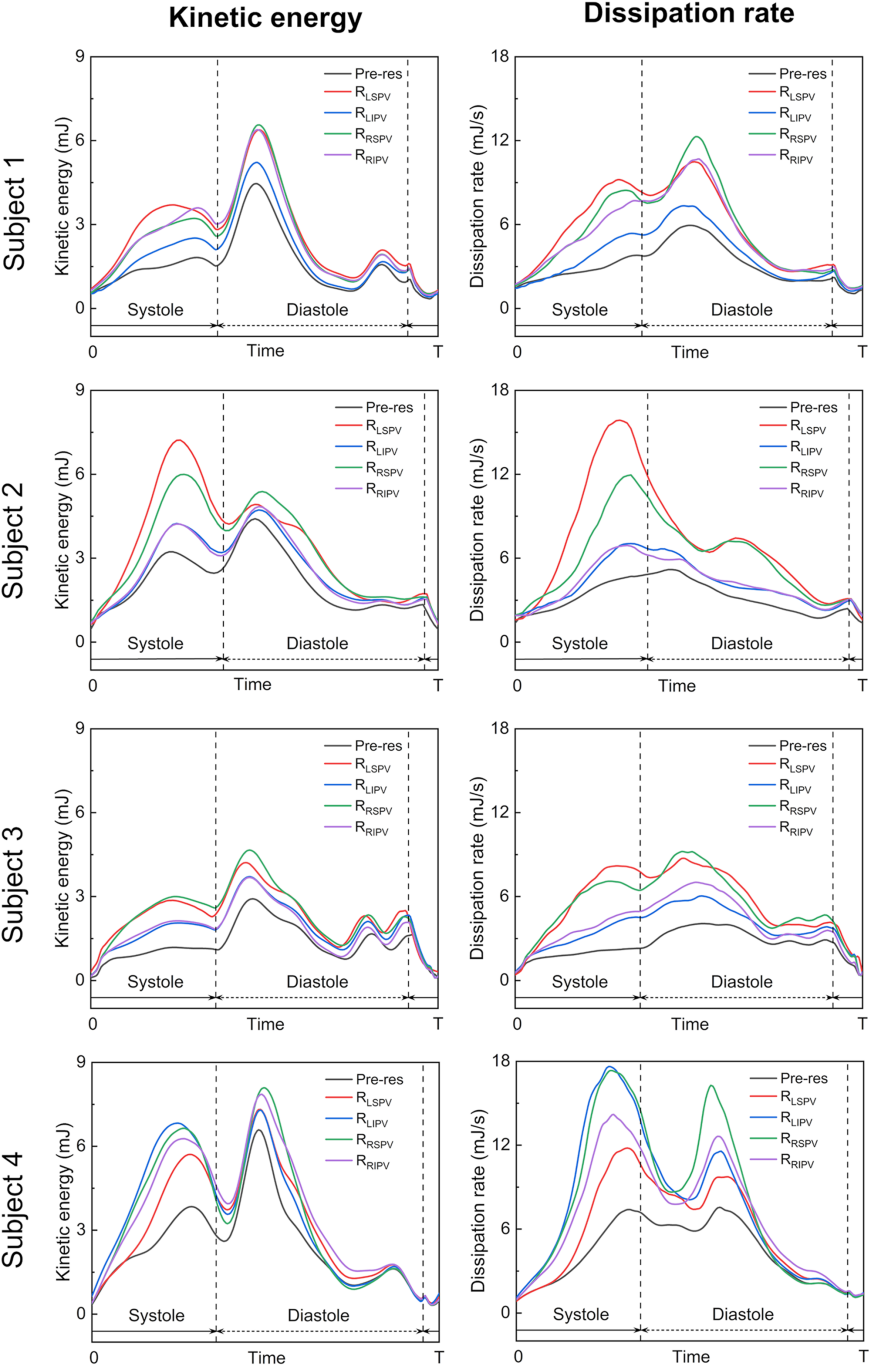
Time courses of the flow kinetic energy (left) and dissipation rate (right) in the left atrium over a whole cardiac cycle in the pre-resection case (Pre-res), left superior pulmonary vein (LSPV) resection case (${\text{R}}_{\text{LSPV}}$), left inferior pulmonary vein (LIPV) resection case (${\text{R}}_{\text{LIPV}}$), right superior pulmonary vein (RSPV) resection case (${\text{R}}_{\text{RSPV}}$), and right inferior pulmonary vein (RIPV) resection case (${\text{R}}_{\text{RIPV}}$), in each participant.

Figure 7 shows time courses of the flow kinetic energy and dissipation rate in the LA before and after each PV resection in each participant. Overall, the kinetic energy and dissipation rate in a whole cardiac cycle showed similar trends in pre-resection and PV resection cases, while in PV resection cases they were relatively larger than the pre-resection ones in all participants. The kinetic energy had three local maximum peaks at mid-systole, early to mid-diastole, and late diastole. In participants 1–3, the kinetic energy increased moderately during the LV systole phase and reached at most 3 mJ in all participants, whereas those in the LSPV resection cases showed the highest values at systole peak compared to other PV resection cases (approximately two to three folds higher than those in pre-resection cases). Also, the dissipation rate in the LV systole phase was almost constant in pre-resection cases (at most 3 mJ/s), whereas those in the LSPV resection case had the highest values at the systolic peak and approached three to four folds higher than those in pre-resection case. On the contrary, the values for LSPV were lower than those for the other PV resections in participant 4, for whom RSPV showed the greatest flow dissipation during a cardiac cycle.

## 4 Discussion

The effect of LUL on LA hemodynamics has received much attention because of its association with LA thrombosis (5, 7). A recent MRI study pointed out the distinctive blood flow patterns in the LA after LUL compared with other lobectomies regardless of thrombus formation (14), but detailed flow characteristics remain elusive because of severe image resolution limitations. Therefore, this study conducted CFD simulations with virtual PV resections and analyzed the resulting LA blood flow patterns.

The blood flow pattern in the LA for each PV resection was visualized using Lagrange descriptors, especially in the upper region of the LA, and the global energetic states were calculated. Before PV resection, the left PV inflow was horizontal and flowed toward the right side of the LA without impingement, whereas both right PV inflows flowed downward in parallel (Figure 3). This asymmetric flow pattern is well known, being shown by existing measurements (25, 26) and CFD simulations (27). As a representative case, in participant 2 in this study, LSPV resection increased LIPV inflow, and the LIPV reached the anterior LA wall with impingement of the right PV inflows causing flow disturbances around the LPSV stump (Figure 5). In contrast, the RSPV resection also enhanced the remaining LSPV inflow, while flow disturbances in the upper LA region were relatively weak, and the effects of LIPV and RIPV resections on the global LA flow patterns were small. These tendencies were also reflected in the global energetic states as relatively strong flow dissipation (Figure 7) in participants 1–3, and these findings are consistent with the clinical observation that LSPV resection tends to form distinctive flow patterns with flow disturbances occurring near the PV stump (14). Because these flow patterns may be induced by asymmetric LA flow patterns originating from anatomically complex LA geometries (25), the LA blood flow alteration after LUL noted above is likely typical in clinical datasets.

However, LA and PV geometries varied markedly among patients, and thus the resulting blood flow patterns also had large variations, as seen in participant 4 in this study. In this case, the LSPV cross-sectional area was the smallest among all PVs (Table 1), and the effects of LSPV resection were smallest on the global energetic state of the LA (Figure 7). In contrast, RSPV resection induced strong flow dissipation, which may have resulted from enhanced flow impingement between the left PV inflows and the remaining RIPV inflows caused by the RSPV resection (Figure 6). Although the LIPV tended to be smallest among all PVs, large patient-specific variations in PV morphologies have been documented (28). Given that PV location and flow rate balance have been considered as factors influencing LA blood flow even for the MV plane and LA appendage (29–31), patient-specific variations in PV morphologies and the remaining PV inflows are essential when considering patient-specific flow characteristics in the PV stump.

It should be noted that this study investigated global LA flow patterns after PV resections, and thus evaluated the flow kinetic energy and dissipation rate in a whole LA domain as indicators of global hemodynamic states. Because the same total flow rate through the LA was set in both pre and post-resection cases based on measurements in our previous studies (15, 16), a PV resection increased the inflow rate and associated flow velocity through the remaining PVs. Furthermore, this inflow enhancement tended to induce flow impingement with viscous dissipation (Figure 5). Therefore, we believe that the relatively high kinetic energy and dissipation rate in post PV resection (Figure 7) are reasonable from a fluid mechanical viewpoint.

This study had four main limitations. First, it aimed to clarify the distinctive LA blood flow patterns after LUL observed in clinical practice, and the association between LA flow patterns and PV stump thrombosis was beyond its scope. Although global LA flow patterns and its flow disturbances are considered one of the risk factors for thrombus formation in the PV stump (7, 9, 14), thrombus formation in PV stump is rare (approximately 5% (5)) depending on several influential factors not only the LA hemodynamics but also PV stump morphologies (7) and physiological factors such as inflammatory responses (12). In addition, due to shortages of patient datasets, four participants recruited in this study are not cases of PV stump thrombosis after LUL. Thus, systematic studies with large patient datasets are necessary to identify the essential factors in PV stump thrombosis. Second, lobectomies may cause physiological changes in the remaining PV inflow, as well as in LA and LV function, as discussed in our previous study (16). Thus, CFD simulation with virtual PV resection may over- or underestimate LA flow alteration after lobectomies. However, comparison of LA flow patterns among PV resections within the same participant is clinically infeasible, and computational modeling is a possible alternative to estimate the effects of each PV resection on LA hemodynamics. Third, this study assumed that the flow velocity was identical among PV inflows and did not consider patient-specific inflow rates. Flow rate balance among PVs is not only determined by the lung lobe volume (32) but is also influenced by body posture (33); therefore, it is difficult to identify even within a single patient. Furthermore, lobectomies lead to expansion of the remaining lung lobes to compensate for the resected lobe function (34). Lobectomies may also alter the inflow rate balance among remaining PV inflows, although to our knowledge no studies have examined PV inflow rate changes after lobectomies. Further clinical investigation of PV inflows would provide valuable insights into the mechanisms underlying the LA blood flow patterns associated with thrombosis. Fourth, quantification of the global LA blood flow patterns by possibly a single parameter is an interesting topic for clinical usage, while these issues include multi-scale turbulent flow dynamics and are still challenging, even in the turbulent research field, to our knowledge. As an alternative approach, we considered visualizing the flow topology from flow velocity fields and extracted the flow boundaries formed by multiple PV inflows. Such postprocessing is effective in interpreting characteristic behaviors of spatiotemporally varying velocity fields while limiting quantitative discussion for further analyses using large patient datasets. For this issue, a data science approach that can handle big data problems would be reasonable to quantify the complex blood flow patterns.

## 5 Conclusions

This study considered the distinctive LA blood flow patterns after LUL pointed out by recent MRI studies relative to those of lobectomies performed in other sites. We performed 4D-CT-based CFD simulations of LA blood flow without and with virtual PV resections, which modeled the effects of lung lobectomy, and compared the blood flow patterns, especially for the remaining PV inflows. LSPV resection tended to enhance LIPV inflow such that it reached the LA anterior wall, resulting in impingement of the right PV inflows in the upper LA region with relatively severe flow disturbances around the PV stump. However, LA flow patterns after PV resections showed large patient-specific variations because of the LA and PV geometries, and the relative effects of each PV resection on the LA flow patterns differed among participants. These results suggest the marked patient-specific variations and highlights the importance of patient-specific assessment of the LA hemodynamics after lobectomies.

## Data Availability

The raw data supporting the conclusions of this article will be made available by the authors, without undue reservation.
